# Probing into the chemopreventive properties of synthetic 1,3,6-tri-O-galloyl-α-D-glucose (α-TGG) against glioblastoma and triple-negative breast cancer-derived cell models

**DOI:** 10.1016/j.crphar.2025.100219

**Published:** 2025-04-05

**Authors:** Carolane Veilleux, Jihane Khalifa, Alain Zgheib, Angélique Sabaoth Konan, Roger Gaudreault, Borhane Annabi

**Affiliations:** a Laboratoire D’Oncologie Moléculaire, Département de Chimie and CERMO-FC, Université du Québec à Montréal, Montréal, QC, H3C 3P8, Canada; b Département de Chimie, Université Du Québec à Montréal, Montréal, QC, H3C 3P8, Canada

**Keywords:** α-TGG, Inflammation, Invasion, EMT, Chemoprevention, Glioblastoma, Breast cancer

## Abstract

Inflammation plays a significant role in cancer progression. Chemopreventive strategies against cellular response to pro-inflammatory cues may therefore contribute to inhibit the acquisition of an invasive phenotype. 1,3,6-Tri-O-Galloyl-β-D-Glucose (β-TGG) is a type of gallotannin naturally found in plants like *Paeonia lactiflora* and *Terminalia chebula.* Unfortunately, the overall yields of β-TGG extraction require complex purification protocols from plant sources and are relatively low. Here, a new synthetic α-anomer of TGG (α-TGG) was characterized for anti-inflammatory and anticancer biological properties. *In vitro* pro-inflammatory and epithelial-to-mesenchymal transition (EMT) cues, triggered by phorbol 12-myristate 13-acetate (PMA), concanavalin A (ConA), tumor necrosis factor (TNF) α, and transforming growth factor (TGF) β, were used to screen α-TGG in two highly aggressive human cancer cell models, namely the U87 glioblastoma and the MDA-MB-231 triple-negative breast cancer (TNBC)-derived cells. α-TGG dose-dependently inhibited ConA-mediated activation of the latent matrix metalloproteinase pro-MMP-2 into its active MMP-2 form as well as the ConA- and PMA-mediated cyclooxygenase (COX)-2 expression, two biomarkers of inflammation, in U87 cells. In MDA-MB-231, α-TGG inhibited PMA- and TNFα-mediated induction of pro-MMP-9, a marker of inflammation and invasive phenotype. Finally, in both cell lines, α-TGG further inhibited TGFβ-induced chemotaxis, as well as TGFβ-induced Smad2 phosphorylation and Snail expression, crucial upstream signaling pathway and downstream biomarkers associated with EMT. Collectively, we confirm that α-TGG retained potent anti-inflammatory and anti-invasive pharmacological properties which support its chemopreventive potential.

## Highlights

• Inflammation plays a significant role in cancer progression.
• Strategies that target pro-inflammatory cues can contribute to inhibit the acquisition of an invasive phenotype.
• 1,3,6-Tri-O-Galloyl-α-D-Glucose (α-TGG) demonstrates *in vitro* anti-inflammatory and anticancer biological properties.
• α-TGG potent anti-inflammatory and anti-invasive pharmacological properties support its chemopreventive potential.

## Graphical abstract

**Figure:**
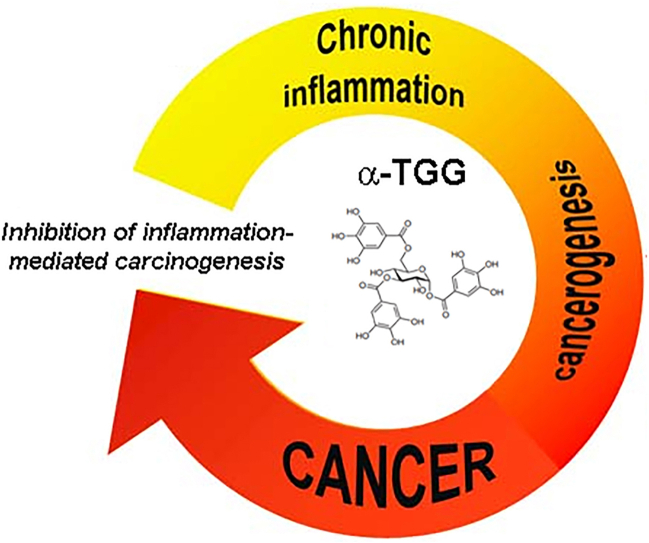

## 1 Introduction

With cancer and its associated chronic inflammation still being a leading cause of death worldwide, prevention becomes as important an avenue of research as treatments are. Preventing inflammation in cancer involves a combination of lifestyle changes and medical interventions (Hou et al., 2021; Todoric et al., 2016). Chronic inflammation creates a tumor-promoting environment through, in part, inflammatory cells infiltration and secretion of mediators within the tumor microenvironment, where they contribute to processes like tissue remodeling and angiogenesis (Demir, 2023). Persistent inflammation can also lead to genomic instability, causing DNA damage, activation of oncogenes, or impairment of tumor suppressor genes (Rajput and Wilber, 2010). Inflammation can further promote the initiation of cancer and facilitate metastasis by interacting with various cellular and non-cellular components in the body (Wen et al., 2022).

Current research actively explores ways to target inflammation as a strategy for both cancer prevention and treatment modalities, with some promising early results (Crusz and Balkwill, 2015; Wang et al., 2024). Many phytochemicals, naturally occurring in fruits, vegetables, and other plant-based foods, can reduce inflammation by targeting various pro-inflammatory processes, such as the inhibition of cytokines production and the enhancement of anti-inflammatory cytokines production (Nisar et al., 2023). Common anti-inflammatory phytochemicals include flavonoids, carotenoids, and polyphenols, which are abundant in foods like berries, citrus fruits, leafy greens, and nuts (Pyo et al., 2024).

Certain phytochemicals found in plants also possess anticancer properties, such as gallotannins, a type of hydrolysable tannin consisting of a central sugar molecule (usually glucose) esterified with multiple gallic acid units (Jourdes et al., 2013). Indeed, many studies on galloyl glucosides, especially the penta-galloyl, showed potent antitumor properties in humans (Li et al., 2015; Wen et al., 2023; Zhang et al., 2009). Multiple bi- and tri-galloyl glucosides had an inhibition rate between 64.2 % and 92.9 % at a concentration of 100 μg/mL on various cancer cell lines, including K562 chronic myeloid leukemia, HL-60 acute promyelocytic leukemia, and HeLa adenocarcinoma cells (Li et al., 2015). Interestingly, recent structure-to-function studies performed on catechins, a polyphenol-type phytochemical, have demonstrated that their galloyl moiety plays a crucial role in enhancing their antioxidant and anti-inflammatory activities (Takahashi et al., 2018), as well as other processes including TGFβ-induced EMT, and inhibition of vasculogenic mimicry (Djerir et al., 2018; Sicard et al., 2021). Computational methods have been further designed for structure-to-function analysis of diet-derived catechins-mediated targeting of *in vitro* vasculogenic mimicry (Uthamacumaran et al., 2021).

The gallotannin 1,3,6-Tri-O-galloyl-β-D-glucose (TGG), or 1,3,6-Tri-O-galloyl-β-D-glucopyranose (NCBI, 2024), is a phytochemical found naturally in plants such as *Paeonia lactiflora* and *Terminalia chebula* (Zhou et al., 2011). Unfortunately, the overall yields of β-TGG purification from plant sources are relatively low due to the complexity of the compound and require elaborate extraction protocols. An improved total synthesis of TGG's α-anomer (α-TGG) was recently reported (Pauvert et al., 2023) which chemopreventive properties have however yet to be characterized. To address whether α-TGG possess any anti-inflammatory or anticancer pharmacological activity, four pro-inflammatory cues (i.e. Phorbol 12-myristate 13-acetate (PMA), concanavalin A (ConA), tumor necrosis factor (TNF) α and transforming growth factor (TGF) β) were used to screen those properties. The experimentations were performed *in vitro* using two highly aggressive human cancer cell models, namely the U87 glioblastoma and the MDA-MB-231 triple-negative breast cancer (TNBC)-derived cells.

α-TGG was found to inhibit, in a dose-dependent manner, the ConA-mediated activation of the latent matrix metalloproteinase (MMP) pro-MMP-2 form into its active MMP-2 form, as well as the ConA- and PMA-mediated cyclooxygenase (COX)-2 expression, two biomarkers of inflammation, in U87 cells. In MDA-MB-231, α-TGG inhibited PMA- and TNFα-mediated induction of pro-MMP-9, a marker of inflammation and invasive phenotype. Finally, in both cell lines, α-TGG further inhibited TGFβ-induced chemotaxis, as well as TGFβ-induced Smad2 phosphorylation and Snail expression, respectively a crucial upstream signaling pathway and a downstream biomarker associated with EMT. Collectively, these anti-inflammatory and anti-invasive pharmacological properties of α-TGG support its chemopreventive potential.

## 2 Materials and methods

### 2.1 Reagents

α-TGG was a kind gift of Yann Pauvert (Université de Montréal, Montreal, QC, Canada) (Pauvert et al., 2023). Zymography standards for pro-MMP-2 and pro-MMP-9 were produced in our own laboratory (Veilleux et al., 2024). PMA, ConA, TNFα, type A gelatin, and protease inhibitor cocktail were purchased from Sigma-Aldrich (Oakville, ON, Canada). Cell culture media were from Wisent (St-Jean-Baptiste, QC, Canada). TGFβ and Pierce's micro bicinchoninic acid (BCA) protein assay reagents were from ThermoFisher Scientific (Waltham, MA, USA). Electrophoresis reagents were purchased from Bio Basic (Markham, ON, Canada). The Clarity™ Enhanced Chemiluminescence (ECL) for HRP (horseradish peroxidase)-conjugated antibody detection reagents were from Bio-Rad (Hercules, CA, USA). The polyclonal antibodies against Snail (3879S), Smad2 (5339S), phospho-Smad2 Ser465/467 (3108S), as well as the monoclonal antibody against Glyceraldehyde-3-phosphate dehydrogenase (GAPDH) (97166S) were from Cell Signaling Technology (Danvers, MA, USA). The polyclonal antibody against COX2 (610203) was from BD Bioscience (Franklin Lake, NJ, USA). HRP-conjugated donkey anti-rabbit and anti-mouse immunoglobulin IgG secondary antibodies were from Jackson ImmunoResearch Laboratories (West Grove, PA, USA).

### 2.2 Cell culture

Human glioblastoma U87 (HTB-14) and TNBC-derived MDA-MB-231 (HTB-26) cells were purchased from the American Type Culture Collection (ATCC; Manassas, VA, USA). U87 cells were cultured in Eagle's Minimum Essential Medium (EMEM) supplemented with 10 % (v/v) fetal bovine serum (FBS; HyClone Laboratories, SH30541.03), 100 units/mL penicillin and 100 mg/mL streptomycin (Wisent, 250-202-EL). MDA-MB-231 cells were cultured in Dulbecco's Modification Eagle's Medium supplemented with 10 % (v/v) FBS, 100 units/mL penicillin, 100 mg/mL streptomycin and 1 % (v/v) non-essential amino acids (HyClone Laboratories, SH40003.01). Both cell lines were cultured in 6-well plates at 3 × 10^5^ cells/mL and incubated in a humidified incubator at 37 °C with 5 % CO_2_. Cells were kept subconfluent and expanded in number over 20 passages by a 1:3–1:4 split, 2 to 3 times per week. Tested compounds, ConA (30 μg/mL), PMA (1 μM), TNFα (30 ng/mL), TGFβ (30 ng/mL) and α-TGG (3–100 μM), were added to serum-free cell culture media, and cells treated for 24 h at 37 °C with 5 % CO_2_.

### 2.3 Zymography

Gelatin zymography was used to assess the extracellular level of gelatinolytic activities of pro-MMP-9, pro-MMP-2, and MMP-2 (Veilleux et al., 2024). In summary, conditioned culture media was harvested and centrifuged at 3500 rpm for 5 min to remove cell debris. A sample of 20 μL was electrophoresed on a gel containing 1 mg/mL gelatin, a substrate that these enzymes are highly effective at hydrolyzing. The gels were incubated in 2.5 % Triton X-100 to renature the MMPs. The gels were washed with ultrapure water before being further incubated at 37 °C overnight in a solution of 200 mM NaCl, 5 mM CaCl_2_, 0.02 % Brij®-35, and 50 mM Tris-HCl buffer, pH 7.6. The gels were then stained with 0.1 % Coomassie Brilliant Blue R-250 in 10 % acetic acid and 40 % methanol in water. Destaining was done in 10 % acetic acid and 30 % methanol in water which allowed detection of gelatinolytic activity as unstained bands on a blue background. Densitometric analysis was performed using ImageJ (Schneider et al., 2012).

### 2.4 Western blot

Total cell lysates were obtained through lysis in a buffer containing 1 mM each of sodium fluoride and sodium orthovanadate as well as 1X proteases inhibitor cocktail. Proteins (10–20 μg) were then separated by SDS-polyacrylamide gel electrophoresis (PAGE). Next, proteins were electro-transferred to low-fluorescence polyvinylidene difluoride (PVDF) membranes and blocked for 1 h at room temperature with 5 % non-fat dry milk in Tris-buffered saline (150 mM NaCl, 20 mM Tris-HCl, pH 7.5) containing 0.3 % Tween-20 (TBST; Bioshop, TWN510-500). Membranes were washed thrice in TBST and incubated overnight with the appropriate primary antibodies (1/1000 dilution) in TBST containing 3 % BSA and 0.1 % sodium azide (Sigma-Aldrich) at 4 °C with agitation. After three washes in TBST, membranes were incubated for 1 h at room temperature with HRP-conjugated anti-rabbit or anti-mouse IgG at 1/2500 dilutions in TBST containing 5 % non-fat dry milk. Immunoreactive bands were visualized by ECL on a Chemidoc or by autoradiography. Densitometric analysis was performed using ImageJ (Schneider et al., 2012).

### 2.5 Real-time cell migration assay

Experiments were carried out using the Real-Time Cell Analysis (RTCA) dual purpose (DP) instrument and the xCELLigence system (Agilent, QC, Canada), following the instructions of the supplier: 25,000 cells per well were seeded in a CIM-plate 16 (Roche Diagnostics) and incubated at 37 °C under a humidified atmosphere containing 5 % CO_2_ for 24 h. Prior to cell seeding, the bottom of each well in the upper chamber was coated with 0.15 % gelatin in phosphate-buffered saline (PBS) and incubated for 1 h at 37 °C. The lower chamber was filled with serum-free medium containing either the vehicle or treatment. The upper chamber of each well was filled with 25,000 cells, in serum-free media as well. After 30 min of initial adhesion, cell migration was monitored for 2–7 h. The impedance value was measured by the RTCA DP instrument and expressed as an arbitrary unit called the Cell Index. Each experiment was performed in quadruplicate wells, data and error bars were expressed as mean ± standard error of the mean (SEM).

### 2.6 Statistical data analysis

Data and error bars were expressed as the mean ± SEM of two to three independent experiments. Hypothesis testing was conducted using a Mann-Whitney *U* test (for comparison of two groups) with probability (P) values of less than 0.05 (∗) considered significant and denoted in the respective figures. All statistical analysis were performed using the GraphPad Prism 7 software version 9.0.0 (GraphPad Software LLC, San Diego, CA, USA).

## 3 Results

### 3.1 *In vitro* pharmacological impact of α-TGG against pro-inflammatory treatments on the gelatinolytic activity and activation status of pro-MMP-2 and pro-MMP-9

**Fig. 1:**
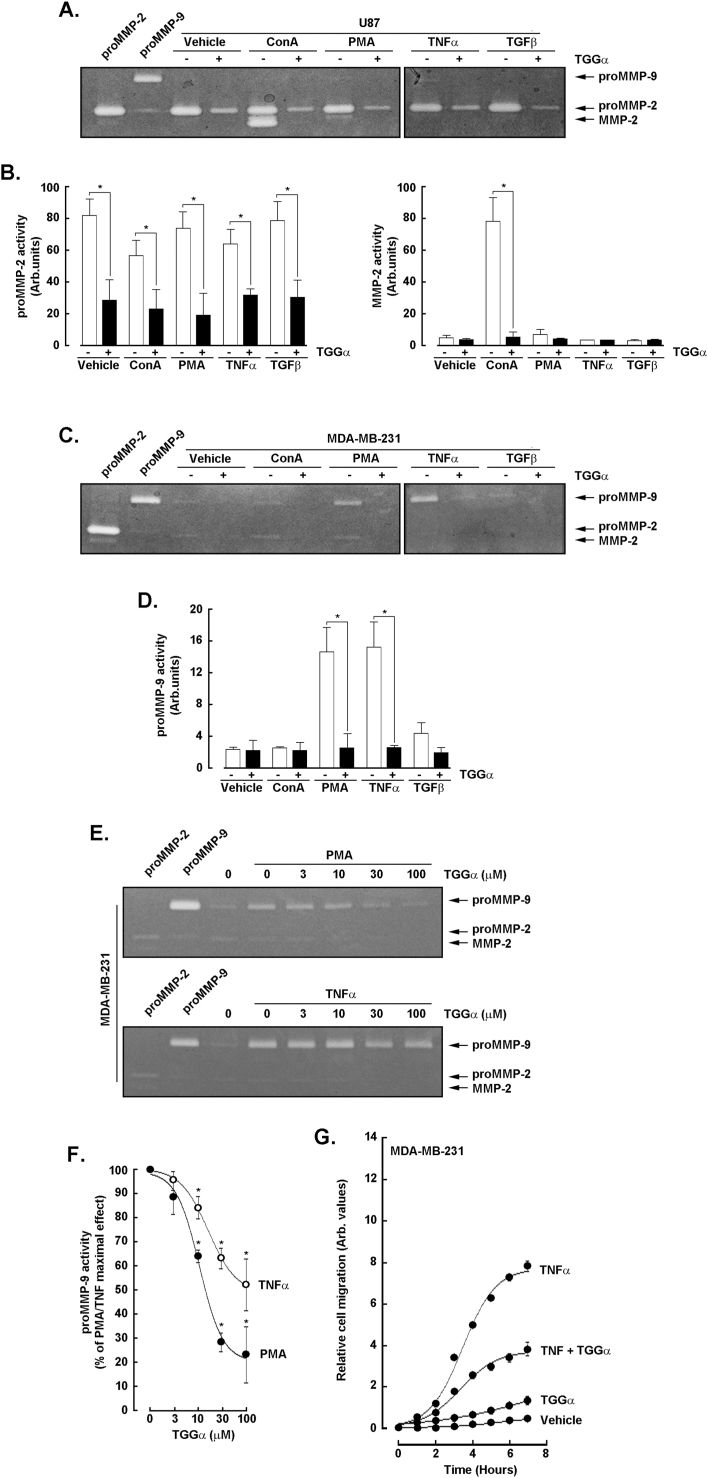
*In vitro pharmacological impact of α-TGG against pro-inflammatory treatments on the secretion and activation status of pro-MMP-2 and pro-MMP-9.* U87 glioblastoma cells and MDA-MB-231 TNBC-derived cells were treated for 24 h with vehicle, 30 μg/ml ConA, 1 μΜ PMA, 30 ng/mL TNFα, or 30 ng/mL TGFβ in serum-free media in the presence or absence of 100 μM α-TGG. Conditioned media were harvested and gelatin zymography performed as described in the Methods section in **A)** U87 cells, **C)** and **E)** MDA-MB-231 cells. A densitometry analysis was performed to assess **B)** the secretion (left panel) and activation levels of pro-MMP-2 (right panel) for the indicated treatment in the presence (black bars) or absence (white bars) of α-TGG in U87 cells, as well as **D)** in MDA-MB-231 cells). **F)** A densitometry analysis was performed to quantify the dose-dependant inhibition by α-TGG of pro-MMP-9 secretion induced by PMA (open circles) and TNFα (closed circles). **G)** Chemotactic real-time cell migration was performed as described in the Methods section on MDA-MB-231 cells only in the presence of vehicle, 100 μM α-TGG, 30 ng/ml TNFα, or a combined α-TGG/TNFα, over a period of 7 h. All data are representative from 2 to 3 independent experiments.

During inflammation, MMPs expression and activity can be induced and contribute to both the progression and resolution of the inflammatory process (O'Shea and Smith, 2014). They are also linked to angiogenesis, cancer growth and metastasis (Klein and Bischoff, 2011). The pro-inflammatory cues of several reputed pharmacological agents including ConA, PMA, TNFα, and TGFβ were therefore assessed in U87 glioblastoma cells as well as in MDA-MB-231 TNBC-derived cells. Media conditioned by the treated cells in the presence or absence of 100 μM α-TGG were harvested and gelatin zymography performed as described in the Methods section (Fig. 1A and C). On the one hand, while very low to undetectable proMMP-9 secretion was observed upon PMA and TNFα treatments in U87 cells, it was significantly induced in MDA-MB-231 cells (Fig. 1D). On the other hand, ConA activated the latent pro-MMP-2 into its active MMP-2 form only in U87 cells (Fig. 1B, right panel). Interestingly, the inclusion of α-TGG further prevented the basal secretion of pro-MMP-2 (Fig. 1B, left panel) and pro-MMP-2 activation in U87 cells (Fig. 1B, right panel), as well as the induction of pro-MMP-9 secretion in MDA-MB-231 cells (Fig. 1D). Altogether, these data suggest that cells were responsive to the pharmacological action of α-TGG against the pro-inflammatory cues tested.

Given the PMA- and TNFα-induced secretion of pro-MMP-9 observed mostly in MDA-MB-231 cells (Fig. 1E) and its importance in cancerous invasion phenotypes (Huang, 2018; Klein and Bischoff, 2011), we further show that α-TGG dose-dependently inhibited PMA- and TNFα-mediated pro-MMP-9 secretion (Fig. 1F). Finally, chemotactic real-time cell migration was also performed with this cell line: TNFα was effectively found to trigger cell migration, whereas α-TGG inhibited such chemotactic cell response (Fig. 1G). Collectively, these data suggest that α-TGG can refrain the acquisition of an invasive phenotype by inhibiting the pro-inflammatory cues, which involve either the activation of latent MMPs, their secretion, chemotactic response, or a combination of these cellular processes.

### 3.2 Dose-dependent inhibition of concanavalin A-mediated activation of pro-MMP-2 by α-TGG in U87 glioblastoma cells

**Fig. 2:**
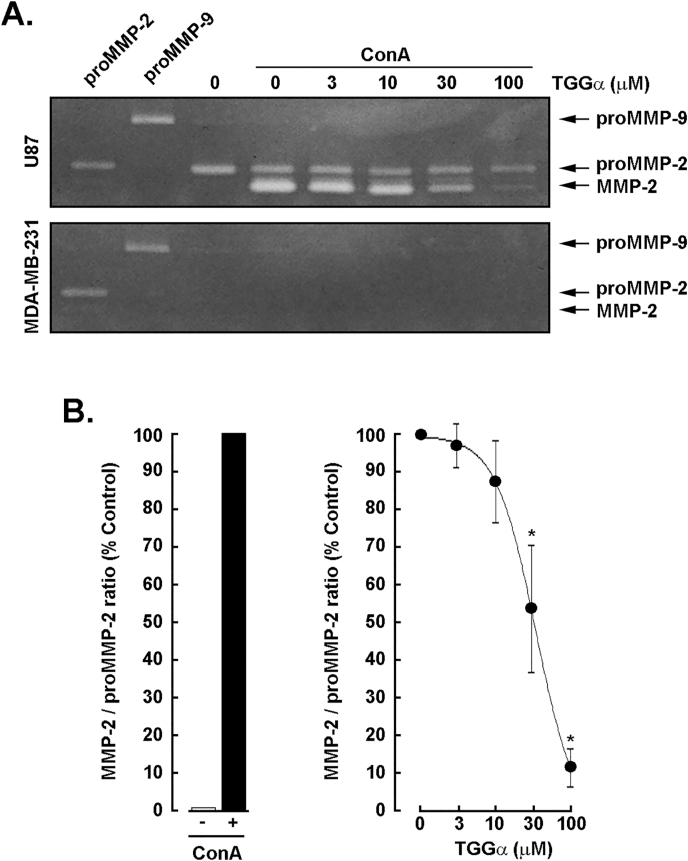
*Dose-dependent inhibition ofconcanavalinA-mediated activation of pro-MMP-*2 by *α-TGG in U87 glioblastoma cells.* U87 glioblastoma cells and MDA-MB-231 TNBC-derived cells were serum-starved and treated for 24 h with 30 μg/ml ConA and increasing concentrations of α-TGG (3–100 μM). **A)** Conditioned media were harvested and gelatin zymography performed as described in the Methods section. **B)** A densitometry analysis was performed to assess the extent of pro-MMP-2 activation into MMP-2 (left panel) and the dose-dependent effect of increasing concentrations of α-TGG (right panel). All data are representative from 2 to 3 independent experiments.

To further explore the impact of α-TGG on the ConA-mediated activation of pro-MMP-2, U87 glioblastoma cells and MDA-MB-231 TNBC-derived cells were serum-starved and treated with 30 μg/mL ConA with increasing concentrations of α-TGG for 24 h. A dose-dependent inhibition of pro-MMP-2 activation was observed when assessed by gelatin zymography (Fig. 2A, upper panel), while very low to undetectable secretion of pro-MMP-2 was observed in MDA-MB-231 cells (Fig. 2A, lower panel). A densitometry analysis confirms the potent pro-MMP-2 activation into MMP-2 (Fig. 2B, left panel) and the dose-dependent inhibition of increasing concentrations of α-TGG which, at 100 μM, almost completely reversed ConA-mediated activation of pro-MMP-2 (Fig. 2B, right panel).

### 3.3 Dose-dependent inhibition of concanavalin A-induced COX-2 expression by α-TGG in U87 glioblastoma cells

**Fig. 3:**
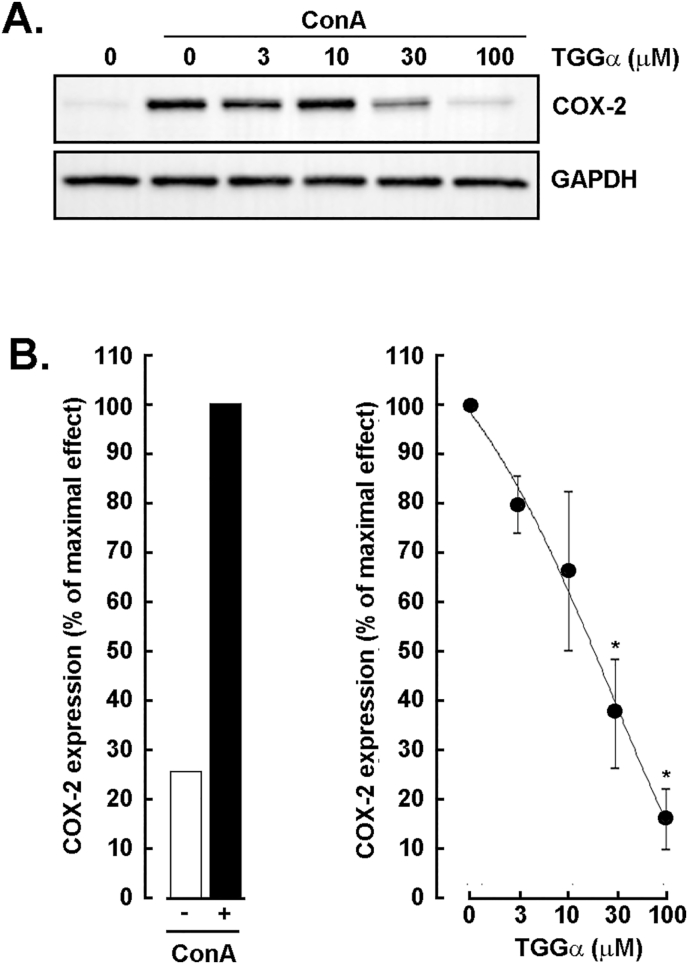
*Dose-dependent inhibition ofconcanavalin A-induced COX-2 expression by α-TGG in U87 glioblastoma cells.* U87 glioblastoma cells were serum-starved and treated for 24 h with 30 μg/ml ConA and increasing concentrations of α-TGG (3–100 μM). **A)** Cell lysates were harvested, and Western blotting performed as described in the Methods section to detect COX-2 expression. GAPDH expression was used as a loading control. **B)** A densitometry analysis was performed to assess the extent of COX-2 expression induced by ConA (left panel) and the dose-dependent effect of increasing concentrations of α-TGG (right panel). Data are representative from 2 independent experiments.

Given the capacity of α-TGG to inhibit ConA-mediated pro-MMP-2 activation, we questioned whether any other intracellular signaling which triggers inflammation may be affected in U87 glioblastoma cells. Cells were treated as described above, protein lysates harvested, and Western blotting performed to detect the pro-inflammatory COX-2 biomarker expression (Fig. 3A, upper panel). ConA effectively induced COX-2 (Fig. 3B, left panel), and α-TGG dose-dependently prevented such induction (Fig. 3B, right panel) as quantified from a densitometry analysis. These data confirm the capacity of α-TGG to alter the signaling cues that lead to the acquisition of a pro-inflammatory phenotype.

### 3.4 Dose-dependent inhibition of PMA-induced COX-2 expression by α-TGG in U87 glioblastoma cells

**Fig. 4:**
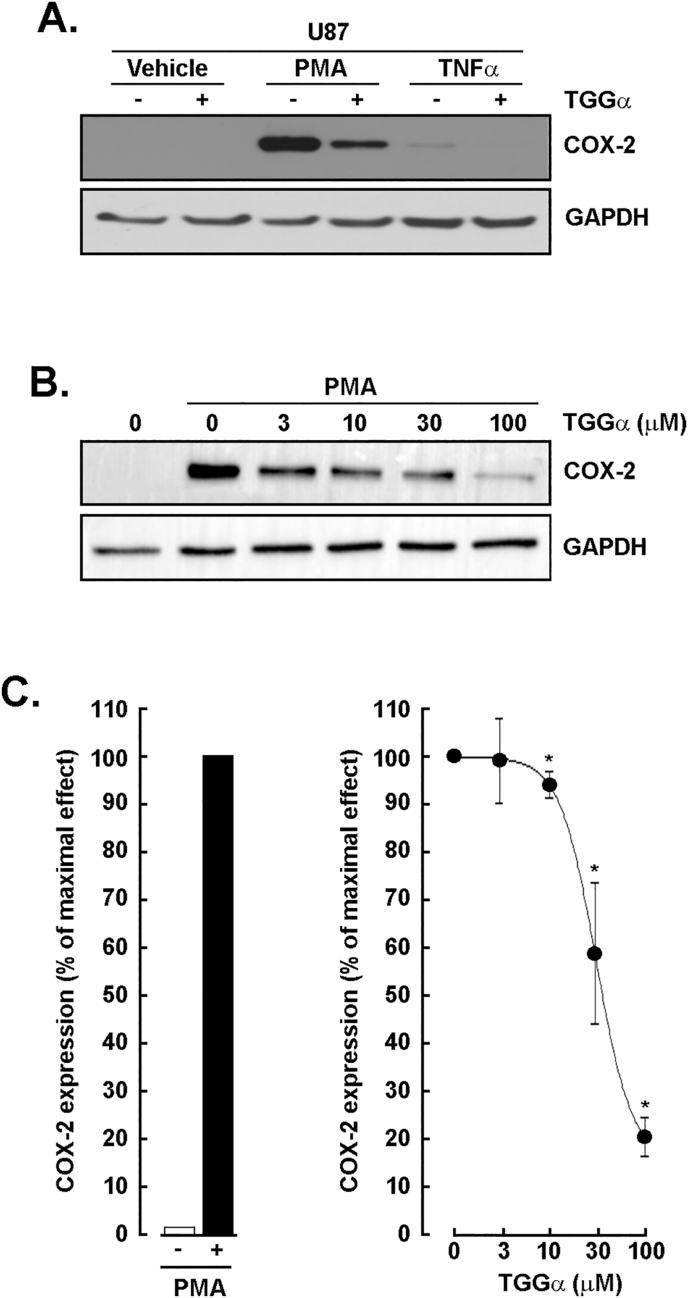
*Dose-dependent inhibition of PMA-induced COX-2 expression by α-TGG in U87 glioblastoma cells.* U87 glioblastoma cells were serum-starved and treated for 24 h with 1 μΜ PMA or 30 ng/mL TNFα in the presence or absence of 100 μM α-TGG. **A)** Cell lysates were harvested, and Western blotting performed as described in the Methods section to detect COX-2 expression. **B)** A representative Western blot of a dose-response effect of α-TGG against PMA-induced COX-2 expression is shown. GAPDH was used as an internal loading control. **C)** A densitometry analysis was performed to assess the extent of COX-2 expression induced by PMA (left panel) and the dose-dependent effect of increasing concentrations of α-TGG (right panel). Data are representative from 2 independent experiments.

We further extended our pharmacological screen to PMA and TNFα, two reputed inducers of inflammation in U87 glioblastoma cells. The cells were treated with either 1 μM PMA or 30 ng/mL TNFα in the presence or absence of α-TGG. Protein lysates were harvested, and Western blotting performed as described in the Methods section to detect COX-2 expression. While TNFα induction was barely detectable, PMA on the other hand significantly triggered COX-2 expression (Fig. 4A), which induction was effectively and dose-dependently reversed by α-TGG (Fig. 4B). A densitometry analysis confirmed the extent of COX-2 expression induced by PMA (Fig. 4C, left panel) and the dose-dependent effect of increasing concentrations of α-TGG (Fig. 4C, right panel). Altogether, this is an additional confirmation of the capacity of α-TGG to alter the impact of pro-inflammatory cues.

### 3.5 Dose-dependent inhibition of TGFβ-induced Smad2 phosphorylation and Snail expression by α-TGG in U87 glioblastoma and MDA-MB-231 cells

**Fig. 5:**
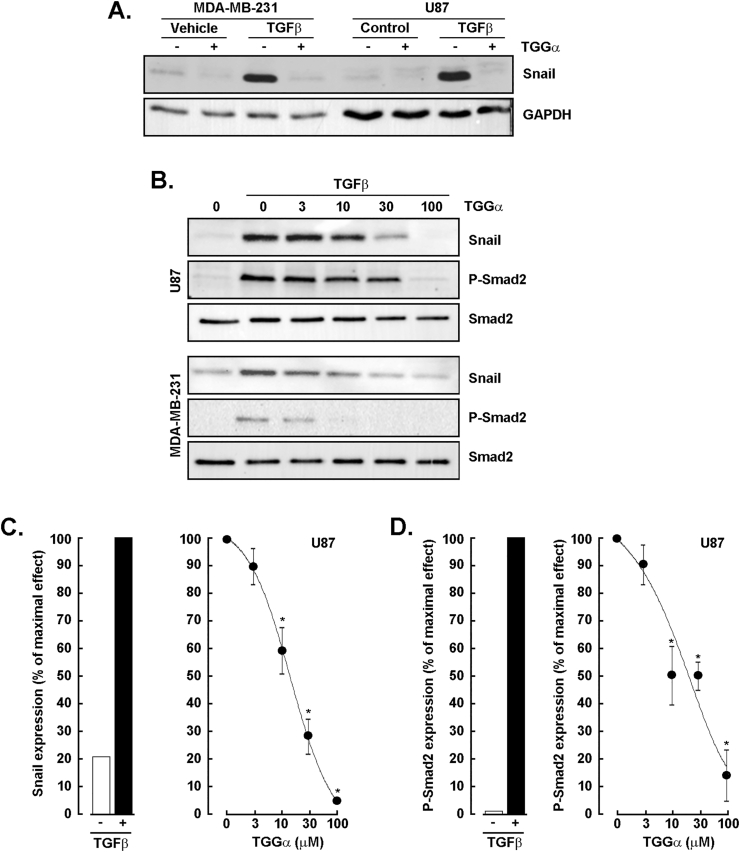
*Dose-dependent inhibition of TGFβ-induced Smad2 phosphorylation and Snail expression by α-TGG in U87 glioblastoma and MDA-MB-231 cells.* U87 glioblastoma and MDA-MB-231 TNBC-derived cells were serum-starved and treated for 24 h with 30 ng/mL TGFβ in the presence or absence of 100 μM α-TGG. **A)** Cell lysates were harvested, and Western blotting performed as described in the Methods section to detect Snail expression. GAPDH was used as an internal loading control. **B)** A representative Western blot of a dose-response effect of α-TGG against TGFβ-induced Snail and phospho-Smad2 expression is shown in U87 glioblastoma cells (upper three panels) and in MDA-MB-231 cells (lower three panels). Smad2 was used as an internal loading control. **C)** A densitometry analysis was performed to assess the extent of Snail expression induced by TGFβ (left panel) and the dose-dependent effect of increasing concentrations of α-TGG (right panel) in U87 glioblastoma cells. **D)** A densitometry analysis was performed to assess the extent of phospho-Smad2 expression induced by TGFβ (left panel) and the dose-dependent effect of increasing concentrations of α-TGG (right panel) in U87 glioblastoma cells. All data are representative from 2 independent experiments.

TGFβ is a pro-inflammatory cytokine also known to trigger the EMT in glioblastoma and ovarian cancer cells (Djediai et al., 2021; Ouanouki et al., 2017; Sicard et al., 2021). U87 glioblastoma and MDA-MB-231 TNBC-derived cells were therefore treated with 30 ng/mL TGFβ and screened in the presence or absence of 100 μM α-TGG. Cell lysates were harvested, and Western blotting performed to detect a significant TGFβ-induced Snail expression which was prevented by α-TGG in both cell lines (Fig. 5A). This inhibitory effect of α-TGG was further found to be dose-dependent in both U87 cells (Fig. 5B, three upper panels) and MDA-MB-231 cells (Fig. 5B, three lower panels) against TGFβ-induced Snail expression as well as Smad2 phosphorylation. A densitometry analysis confirmed the extent of Snail expression induced by TGFβ (Fig. 5C, left panel) and the dose-dependent effect of increasing concentrations of α-TGG (Fig. 5C, right panel) in U87 cells. The extent of TGFβ-mediated phosphorylation of Smad2 (Fig. 5D, left panel) was also found to be dose-dependently inhibited by increasing concentrations of α-TGG (Fig. 5D, right panel). Similar inhibitory profiles were obtained with MDA-MB-231 cells (Fig. 5B, lower panels). Here, evidence suggests that, along with its anti-inflammation properties, α-TGG can also alter the acquisition of an invasive TGFβ-mediated EMT phenotype in two cell models.

### 3.6 α-TGG inhibits the TGFβ-induced chemotactic cell response in U87 glioblastoma and MDA-MB-231 cells

**Fig. 6:**
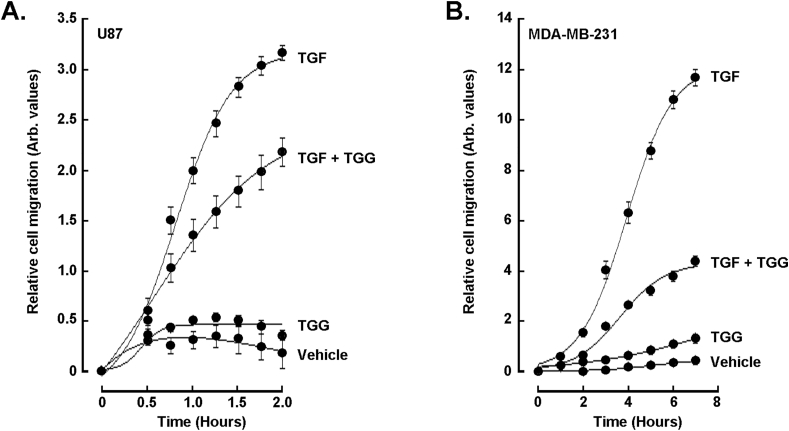
*α-TGG inhibits the TGFβ-induced chemotactic cell response in U87 glioblastoma and MDA-MB-231 cells.* Real time chemotactic cell migration was assessed as described in the Methods section in **A)** U87 glioblastoma and **B)** MDA-MB-231 TNBC-derived cells, in response to vehicle, 30 ng/mL TGFβ, 100 μM α-TGG, or a combination of α-TGG/TGFβ, over a period of 2 and 7 h respectively. Data are triplicates from a representative experiment for each cell line.

**Supplemental Fig. S1:**
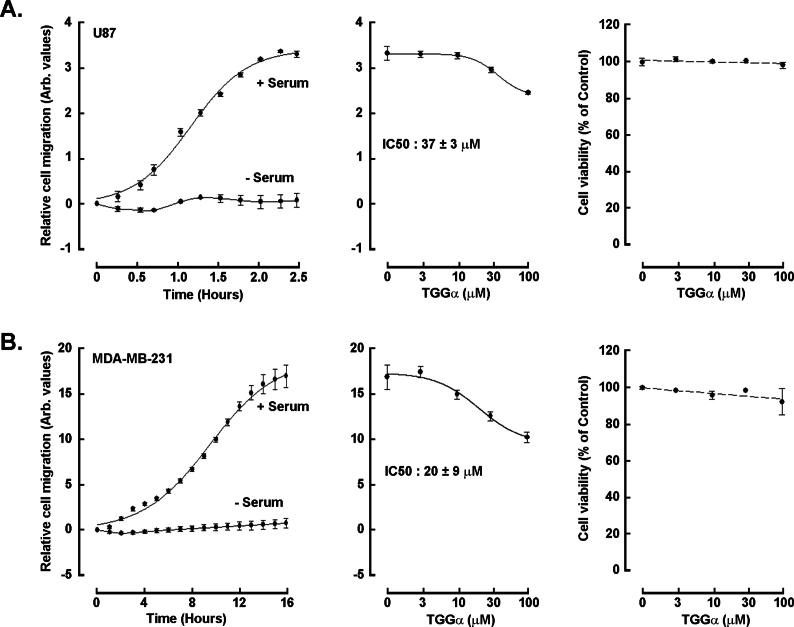
*α-TGG inhibits serum-induced chemotactic cell response in U87 glioblastoma and MDA-MB-231 cells.* Real time chemotactic cell migration was assessed as described in the Methods section in **A)** U87 glioblastoma or **B)** MDA-MB-231 TNBC-derived cells, in response to serum or serum-free media (left panels), and in the presence of increasing concentrations of α-TGG (3–100 μM) (middle panels) over 7 h. Cell viability was also evaluated for each concentration of α-TGG in negative media using the Trypan Blue exclusion assay and a TC20 Automated Cell Counter (Biorad; right panels). Data are representative from two independent experiments.

We finally assessed whether the real-time chemotactic cell migration response to TGFβ was also altered by α-TGG. In accordance with their molecular response to TGFβ documented above, migration of U87 glioblastoma (Fig. 6A, left panel) and MDA-MB-231 TNBC-derived (Fig. 6B) cells was found to be induced by TGFβ and significantly reduced by α-TGG. Interestingly, chemotactic cell migration was also found to be inhibited by α-TGG in response to serum-enriched media (Supplemental Fig. S1). Moreover, such inhibition was not attributed to cytotoxic effects of α-TGG as assessed by the common method of Trypan Blue exclusion assay to determine cell viability (Shekhawat et al., 2025).

## 4 Discussion

Pharmacological probing into the anti-inflammatory mechanisms of action is among the initial essential molecular steps for developing comprehensive cancer treatment and prevention strategies. Given the link between chronic inflammation and cancer where inflammatory cues can promote tumor growth, metastasis, and resistance to treatment (Turizo-Smith et al., 2024), pharmacological screening strategies for new molecules with potential anti-inflammatory and anticancer properties can provide invaluable information in early detection and prevention by identifying the expression profiles of inflammatory biomarkers and invasive processes. Here, we specifically explored the anti-inflammatory properties of α-TGG to reduce tumor-related inflammation, which understanding may improve the effectiveness of its use in chemopreventive and therapeutic modalities.

Studies on galloyl glucosides did show that multiple gallotannins possessed anticancer properties (Li et al., 2015; Wen et al., 2023; Zhang et al., 2009). However, although the TGG properties are yet to be characterized, its potential therapeutic effects have been recognized against ferroptosis (Li et al., 2020) as well as in Alzheimer's disease (Khalifa et al., 2023) and SARS-CoV-2 (Binette et al., 2021; Haddad et al., 2022). An improved method for TGG synthesis was recently developed and allowed the generation of a new α-anomer of TGG (α-TGG) (Pauvert et al., 2023). Here, α-TGG was tested for its ability to target specific mechanisms and pathways which can potentially further reduce inflammation and the risk of cancer recurrence and metastasis (Joshi et al., 2024). A critical step in developing robust and comprehensive cancer therapies implies testing anticancer molecules against highly aggressive and resistant to treatment cancer cells as exemplified in this study using U87 glioblastoma and MDA-MB-231 TNBC-derived cell models. This sheds light for a better understanding of drug efficacy, and to better clinical outcome prediction to guide future clinical trial designs.

The first molecular aspect investigated here was the impact of α-TGG on MMPs as these enzymes play a crucial role in remodeling of the extracellular matrix (ECM), in the regulation of the inflammatory response, in angiogenesis, cancer growth and metastasis (Klein and Bischoff, 2011; Lagente et al., 2011; Löffek et al., 2011; O'Shea and Smith, 2014). Inflammatory cues were used, starting with ConA, a plant-derived lectin known to trigger pro-inflammatory and immune responses in various immune cells through the release of pro-inflammatory cytokines like IL-1β, IL-6, and TNFα (Cai et al., 2024; Mohamed et al., 2022; Zhuang et al., 2016). ConA also induces the Membrane Type (MT)1-MMP, or MMP-14, which in turns cleave the pro-domain of the latent pro-MMP-2 form (Akla et al., 2012; Nanni et al., 2016). This ConA-mediated activation of MMP-2 was indeed observed by zymography in the U87 glioblastoma cell model, and α-TGG inhibited this activation. It is yet unclear whether such action was directed toward the secretion/activation of pro-MMP-2, or the catalytic activity of MMP-2 itself. α-TGG also inhibited ConA-induced expression of COX-2, a pro-inflammatory biomarker requiring the NFκB signaling pathway (Sina et al., 2010; Zhao et al., 2021).

The second pro-inflammatory cue used was TNFα, a pleiotropic cytokine that can lead to chronic inflammation associated with various autoimmune diseases and inflammatory conditions (Alim et al., 2024). Our data showed the induction of pro-MMP-9 by TNFα in MDA-MB-231 cells and, concomitantly, the induction of cell chemotaxis. Both effects were inhibited dose-dependently by α-TGG, thus demonstrating anti-invasive effects as pro-MMP-9 is associated with an invasive cancerous phenotype with increased cell migration, metastasis and angiogenesis (Huang, 2018). The same results were obtained for PMA, a molecule that can activate Protein Kinase C (PKC), which in turn can lead to the expression of various pro-inflammatory cytokines including IL-1β, IL-2, IL-6, IL-8, and TNFα in different cell types, such as T cells and macrophages (Patil et al., 2015). Similarly, PMA effects were found inhibited by α-TGG, confirming its anti-inflammatory properties.

The last pro-inflammatory cue used was TGFβ, a complex cytokine with both pro-inflammatory and anti-inflammatory roles (Giarratana et al., 2024; Yoshimura et al., 2010). It is well-known for its capacity to trigger the EMT, which is characterized by an increase in cell invasion (Sicard et al., 2021). Our data showed that TGFβ induced the phosphorylation of Smad2 and the expression of Snail as well as chemotaxis in both cell lines, and that α-TGG inhibited these effects in a dose-dependent manner, showing anti-invasion properties. Finally, Li et al. (2015) reported that many galloyl glucosides molecules had an inhibition rate between 64.2 % and 92.9 % at a concentration of 100 μg/mL with an IC_50_ ranged from 17.2 to 124.7 μg/mL, on various cancer cell lines. This correlates with our findings as the maximum concentration used in our study was 100 μM, which corresponds to 63.7 μg/mL, and it showed an almost complete reversal for most experiments. Finally, an interesting parallel can be done between α-TGG and 1,2,3,4,6-penta-O-galloyl-D-glucopyranose (PGG), a gallotannin renowned for its significant anti-inflammatory and anti-cancer properties, where the α-anomer was found to stand out as more potent than the β-anomer. Subsequent research into α-PGG has demonstrated its powerful pro-apoptotic effects on human colon carcinoma cell lines (Cao et al., 2011; Li et al., 2005).

## 5 Conclusion

Chemoprevention is a very important field of cancer research, and numerous and varied phytochemicals have shown great promise in that regard. Many have anticancer properties, just as we discovered for the gallotannin α-TGG. It showed pharmacological anti-inflammatory and anti-invasive effects on two aggressive *in vitro* cancer cell models, the U87 glioblastoma and MDA-MB-231 TNBC cells, against four different pro-inflammatory cues (ConA, PMA, TNFα and TGFβ). Indeed, we report here that α-TGG inhibited both the secretion and activation of two MMPs as well as the expression of inflammation and EMT protein biomarkers. It also reduced the chemotaxis effect on cell migration upon pro-inflammatory cues.

This research hints at the very interesting anticancer properties of α-TGG. *In vivo* preclinical studies will be required to dive deeper into its full effects and further decipher its mechanisms of action. However, it would also be interesting to test α-TGG in other contexts than cancer as its β-anomer showed potential as a therapeutic agent for diseases, like Alzheimer's and SARS-CoV-2, as well as an antiferroptosis agent (Binette et al., 2021; Khalifa et al., 2023; Li et al., 2020).

## CRediT authorship contribution statement

**Carolane Veilleux:** Conceptualization, Data curation, Formal analysis, Investigation, Methodology, Writing – original draft, Writing – review & editing. **Jihane Khalifa:** Data curation, Formal analysis, Methodology. **Alain Zgheib:** Data curation, Methodology. **Angélique Sabaoth Konan:** Methodology. **Roger Gaudreault:** Formal analysis, Supervision, Writing – review & editing. **Borhane Annabi:** Conceptualization, Formal analysis, Funding acquisition, Supervision, Writing – original draft, Writing – review & editing, All authors read and approved the final manuscript.

## Data availability

The data used to support the findings of this study are included in the manuscript.

## Funding

This work was funded by a grant from the Natural Sciences and Engineering Research Council of Canada (NSERC, RGPIN-2024-04541).

## Declaration of competing interest

The authors declare that they have no known competing financial interests or personal relationships that could have appeared to influence the work reported in this paper.

## Data Availability

Data will be made available on request.

## Abbreviations

ATCC, American type culture collection; BSA, Bovine serum albumin; ConA, Concanavalin A; COX-2, Cyclooxygenase-2; DMEM, Dulbecco's Modified Eagle Medium; ECL, Enhanced chemiluminescence; ECM, Extracellular matrix; EMEM, Eagle's Minimum Essential Medium; EMT, Epithelial-to-mesenchymal transition; FBS, Fetal bovine serum; GAPDH, Glyceraldehyde-3-phosphate dehydrogenase; HRP, Horseradish peroxidase; IL, interleukin; MMP, Matrix metalloproteinase; PAGE, Polyacrylamide gel electrophoresis; PBS, phosphate-buffered saline; PMA, Phorbol 12-myristate 13-acetate; PVDF, Polyvinylidene difluoride; RTCA DP, Real-Time Cell Analysis dual purpose; SDS, Sodium dodecyl sulfate; SEM, Standard error of the mean; TBST, Tris-buffered saline + Tween-20; TGFβ, Transforming growth factor beta; α-TGG, 1,3,6-Tri-O-galloyl-α-D-glucose; TNBC, Triple-negative breast cancer; TNFα, Tumor necrosis factor alpha; v/v, volume by volume.
